# Dynamic trajectory of platelet-related indicators and survival of severe COVID-19 patients

**DOI:** 10.1186/s13054-020-03339-x

**Published:** 2020-10-14

**Authors:** Jieyu He, Yongyue Wei, Jiao Chen, Feng Chen, Wei Gao, Xiang Lu

**Affiliations:** 1 Department of Epidemiology and Biostatistics, School of Public Health, Center for Global Health, Nanjing Medical University, Nanjing, 211166 China; 2 Department of Geriatrics, Sir Run Run Hospital, Nanjing Medical University, 109 Longmian Avenue, Nanjing, 211166 China

Dear Editor,

Previous studies have found approximately a 30% cumulative incidence for thrombosis in critically unwell patients, almost all whom already present impaired platelets function and activity, with COVID-19 in the intensive care unit (ICU) [1, 2]. We aimed to explore the association between platelet-related laboratory indicators and prognosis in critically ill patients with COVID-19.

All the severe and critically ill COVID-19 patients (Table 1) diagnosed in Huangshi City, Hubei Province, China, till 6 March, 2020, were recruited in this study which were distributed in the three hospitals including Huangshi Central Hospital, Huangshi Hospital of Traditional Chinese Medicine, and Daye People’s Hospital. Laboratory examinations including routine blood tests, lymphocyte subsets, inflammatory or infection-related biomarkers, cardiac, renal, liver and coagulation function tests were obtained at admission and during hospitalization. The baseline laboratory measures with over 40% missing value were excluded from the analysis. Death in 28 days after admission to the hospital was the primary end point of this study. Patients discharge from hospital within 28 days or kept in hospitalization after 28 days were considered as censored outcome. Time-to-event outcome was defined for the following statistical models.

**Table 1:** Demographic and clinical characteristics at hospitalization of severe or critically ill COVID-19 patients

Characteristics	*N*_Missing_ (%)	Total (*n* = 112)	Survived (*n* = 81)	Dead (*n* = 31)
Age [mean (SD)]		61.0 (14.9)	57.1 (13.8)	71.0 (13.0)
Male [*n* (%)]		73 (65.2)	54 (66.7)	19 (61.3)
Vital signs [mean (SD)]				
Temperature (°C)	2 (1.8)	37.3 (0.8)	37.3 (0.8)	37.2 (0.8)
Heart rate (beats/min)	29 (25.9)	89.4 (17.7)	87.1 (16.7)	94.4 (19.0)
Respiratory rate (Breaths/min)	5 (4.5)	24.8 (5.6)	25.1 (5.9)	24.1 (4.9)
Blood pressure (mm Hg)				
Diastolic	5 (4.5)	73.2 (13.7)	73.3 (14.7)	72.8 (11.0)
Systolic	5 (4.5)	124.9 (17.3)	124.0 (18.0)	127.0 (15.7)
Symptoms [*n* (%)]				
Fever		91 (81.2)	67 (82.7)	24 (77.4)
Cough		86 (76.8)	62 (76.5)	24 (77.4)
Chest tightness		73 (65.2)	56 (69.1)	17 (54.8)
Fatigue		65 (58.0)	54 (66.7)	11 (35.5)
Shortness of breath		34 (30.4)	21 (25.9)	13 (41.9)
Phlegm		28 (25.0)	20 (24.7)	8 (25.8)
Dyspnea		25 (22.3)	14 (17.3)	11 (35.5)
Diarrhea		19 (17.0)	15 (18.5)	4 (12.9)
Headache		9 (8.0)	7 (8.6)	2 (6.5)
Myalgia		6 (5.4)	5 (6.2)	1 (3.2)
Sore throat		5 (4.5)	4 (4.9)	1 (3.2)
Nausea and vomiting		5 (4.5)	2 (2.5)	3 (9.7)
Imaging abnormality^a^		18 (16.1)	13 (16.0)	5 (16.1)
No. of symptoms [*n* (%)]				
0		2 (1.8)		2 (6.5)
1		4 (3.6)	4 (4.9)	
2		15 (13.4)	10 (12.3)	5 (16.1)
3		20 (17.9)	15 (18.5)	5 (16.1)
4		30 (26.8)	23 (28.4)	7 (22.6)
5		23 (20.5)	16 (19.8)	7 (22.6)
6		12 (10.7)	8 (9.9)	4 (12.9)
≥ 7		6 (5.4)	5 (6.2)	1 (3.2)
Comorbidities [*n* (%)]				
Hypertension		40 (35.7)	26(32.1)	14 (45.2)
Respiratory failure		27 (24.1)	16 (19.8)	11 (35.5)
Cardiovascular disease		17 (15.2)	10 (12.3)	7 (22.6)
Diabetes		21 (18.8)	15 (18.5)	6 (19.4)
Acute lung injury		14 (12.5)	9 (11.1)	5 (16.1)
COPD^b^		5 (4.5)	2 (2.5)	3 (9.7)
Bacterial pneumonia		3 (2.7)	2 (2.5)	1 (3.2)
Hepatic injury		3 (2.7)	3 (3.7)	
Septic shock		3 (2.7)	2 (2.5)	1 (3.2)
Cerebral infarction		2 (1.8)	1 (1.2)	1 (3.2)
Acute kidney injury		1 (0.9)	1 (1.2)	
Cerebral hemorrhage		1 (0.9)	1 (1.2)	
Sepsis		1 (0.9)	1 (1.2)	
*N* of comorbidities [*n* (%)]				
0		46 (41.1)	36 (44.4)	10 (32.3)
1		26 (23.2)	20 (24.7)	6 (19.4)
2		19 (17.0)	13 (16.0)	6 (19.4)
3		13 (11.6)	7 (8.6)	6 (19.4)
4		4 (3.6)	2 (2.5)	2 (6.5)
5		1 (0.9)	1 (1.2)	0 (0)
≥ 6		3 (2.7)	2 (2.5)	1 (3.2)
Worst severity in hospital				
Severe		63	63	0
Critical illness [*n* (%)]		49	18	31

*SD* standard deviation

^a^Including chest radiography and computed tomography (CT)

^b^Chronic obstructive pulmonary disease

The platelet-related indicators included platelet count (PLT), mean platelet volume (MPV), platelet distribution width (PDW), thrombocytocrit (PCT), and platelet large cell ratio (P-LCR). Baseline indicators were dichotomized by the median to low and high groups. For each platelet-related indicator with repeated examinations during hospitalization, trajectory analysis was performed to cluster the patients based on the dynamic time-series trend of the corresponding indicator, using R package *traj* [3]. According to the requirement of the method, patients during hospitalization with less than four observations of the specific indicator were classified as a separate cluster. Cox proportional hazards model with adjustment for age, gender, number of comorbidities were applied to test the association between dynamic trajectory of platelet-related indicators and overall survival of COVID-19 patients.

The patients at admission with high PLT (HR 0.28; 95% CI 0.11–0.69; *P* = 0.0057; Fig. 1a) were associated with the preferred survival; however, patients with high PDW (HR 2.52; 95% CI 1.17–5.44; *P* = 0.0185; Fig. 1b), high MPV (HR 3.73; 95% CI 1.55–9.02; *P* = 0.0034; Fig. 1c), or high P-LCR (HR 3.00; 95% CI 1.40–6.41; *P* = 0.0046; Fig. 1d) were significantly associated with the worse survival. On the other hand, dynamic trajectory of PLT couldn’t distinguish patients’ survival (Fig. 1e). However, a similar dynamic trajectory pattern with rapid acceleration in the first 2 weeks followed by a considerable deceleration, was identified for MPV, PLCR, and PDW; patients with such pattern were significantly associated with about 2 to 5 times increased death hazard (Fig. 1f–h). All the above results remained significant after false discovery rate (FDR) control.

**Fig. 1:**
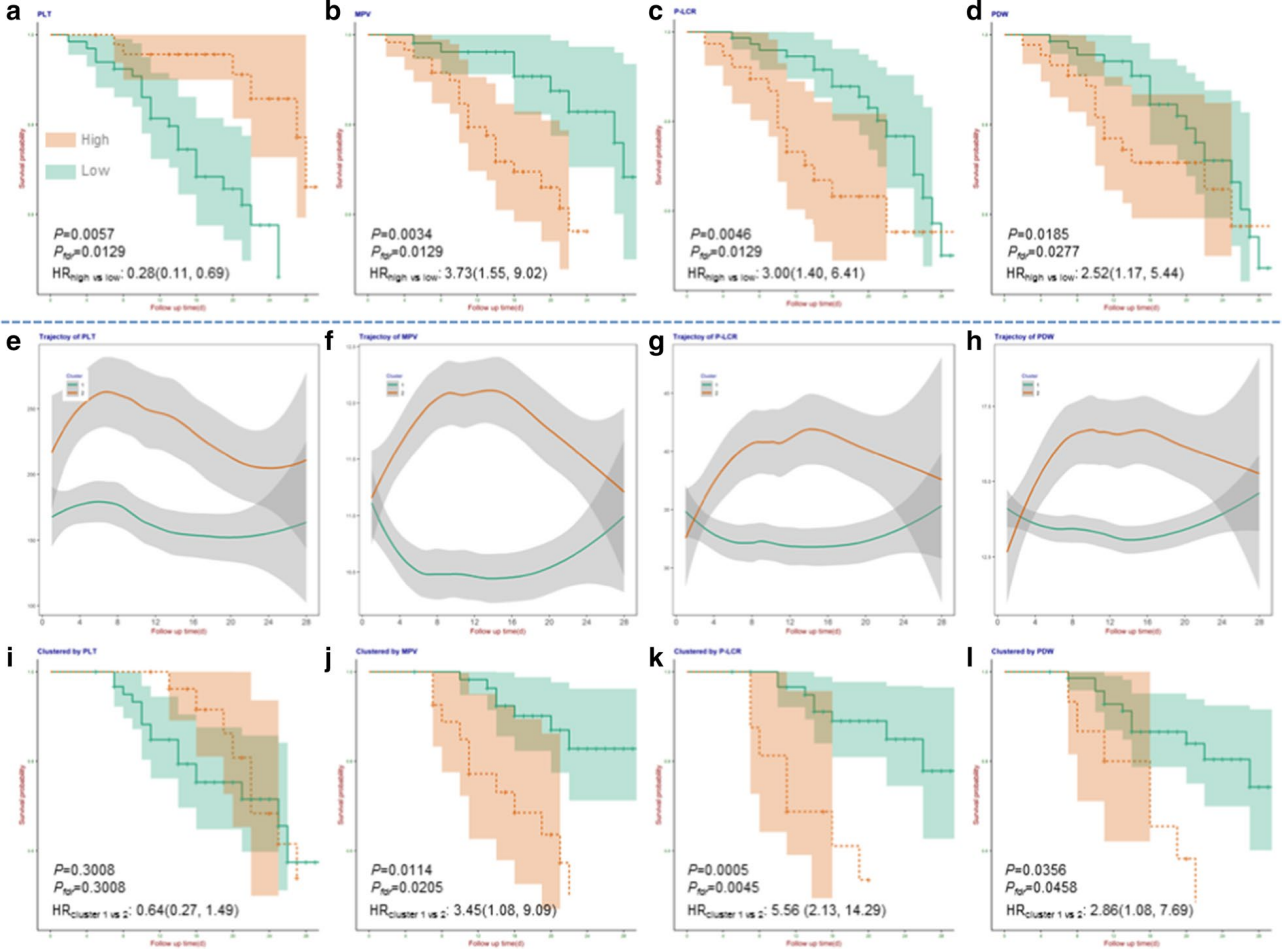
Platelet-related indicators and their dynamic changes that associated with prognosis of severe or critically ill COVID-19 patients. **a** association between baseline platelet count (PLT) and prognosis of patients; **b** association between baseline mean platelet volume (MPV) and prognosis of patients; **c** association between baseline platelet large cell ratio (P-LCR) and prognosis of patients; **d** association between baseline platelet distribution width (PDW) and prognosis of patients; **e** trajectory of PLT; **f** trajectory of MPV; **g** trajectory of P-LCR; **h** trajectory of PDW; **i** association between trajectory of PLT and prognosis of patients; **j** association between trajectory of MPV and prognosis of patients; **k** association between trajectory of P-LCR and prognosis of patients; **l** association between trajectory of PDW and prognosis of patients. Thrombocytocrit (PCT) was not significant after false discovery rate control (*P* = 0.0545), and the trajectory of PCT was not available because the majority of patients lacked follow-up nodes

The findings of this study were accordant with several evidences suggesting platelets as well as related indicators participating in inflammation and prothrombotic responses in many viral infections [4]. The damage to endothelial cells leads to activation, aggregation, and retention of platelets, and the formation of thrombus at the injured site, which may cause a depletion of platelets and megakaryocytes, resulting in decreased platelets production and increased consumption. In addition to their traditional role in thrombosis and hemostasis, platelets mediate key aspects of inflammatory and immune processes [5]. Platelets have been reported to express surface receptors able to mediate binding and entry of various viruses [6]. In brief, paying close attention to the dynamics of platelet-related indicators of COVID-19 patients will undoubtedly improve our knowledge on diseases progression, but could also bring the improvement in therapeutic options for severe or critically ill patients.

## Data Availability

Dr. X. Lu had full access to all of the data in the study. After publication, the data will be made available to others on reasonable requests after approval from the author (luxiang66@njmu.edu.cn).
